# Deep Learning Enables Prostate MRI Segmentation: A Large Cohort Evaluation With Inter-Rater Variability Analysis

**DOI:** 10.3389/fonc.2021.801876

**Published:** 2021-12-21

**Authors:** Yongkai Liu, Qi Miao, Chuthaporn Surawech, Haoxin Zheng, Dan Nguyen, Guang Yang, Steven S. Raman, Kyunghyun Sung

**Affiliations:** ^1^ Department of Radiological Sciences, David Geffen School of Medicine, University of California, Los Angeles, CA, United States; ^2^ Physics and Biology in Medicine Interdisciplinary Program (IDP), David Geffen School of Medicine, University of California, Los Angeles, CA, United States; ^3^ Department of Radiology, The First Affiliated Hospital of China Medical University, Shenyang City, China; ^4^ Department of Radiology, Division of Diagnostic Radiology, Faculty of Medicine, Chulalongkorn University and King Chulalongkorn Memorial Hospital, Bangkok, Thailand; ^5^ Department of Computer Science, Henry Samueli School of Engineering and Applied Science, University of California, Los Angeles, CA, United States; ^6^ Medical Artificial Intelligence and Automation Laboratory, Department of Radiation Oncology, University of Texas Southwestern Medical Center, Dallas, TX, United States; ^7^ National Heart and Lung Institute, Imperial College London, London, United Kingdom

**Keywords:** prostate segmentation, deep attentive neural network, large cohort evaluation, qualitative evaluation, quantitative evaluation, volume measurement

## Abstract

Whole-prostate gland (WPG) segmentation plays a significant role in prostate volume measurement, treatment, and biopsy planning. This study evaluated a previously developed automatic WPG segmentation, deep attentive neural network (DANN), on a large, continuous patient cohort to test its feasibility in a clinical setting. With IRB approval and HIPAA compliance, the study cohort included 3,698 3T MRI scans acquired between 2016 and 2020. In total, 335 MRI scans were used to train the model, and 3,210 and 100 were used to conduct the qualitative and quantitative evaluation of the model. In addition, the DANN-enabled prostate volume estimation was evaluated by using 50 MRI scans in comparison with manual prostate volume estimation. For qualitative evaluation, visual grading was used to evaluate the performance of WPG segmentation by two abdominal radiologists, and DANN demonstrated either acceptable or excellent performance in over 96% of the testing cohort on the WPG or each prostate sub-portion (apex, midgland, or base). Two radiologists reached a substantial agreement on WPG and midgland segmentation (*κ* = 0.75 and 0.63) and moderate agreement on apex and base segmentation (*κ* = 0.56 and 0.60). For quantitative evaluation, DANN demonstrated a dice similarity coefficient of 0.93 ± 0.02, significantly higher than other baseline methods, such as DeepLab v3+ and UNet (both p values < 0.05). For the volume measurement, 96% of the evaluation cohort achieved differences between the DANN-enabled and manual volume measurement within 95% limits of agreement. In conclusion, the study showed that the DANN achieved sufficient and consistent WPG segmentation on a large, continuous study cohort, demonstrating its great potential to serve as a tool to measure prostate volume.

## Introduction

Whole-prostate gland (WPG) segmentation plays an important role in prostate volume measurement, biopsy, and surgical planning (1). Magnetic resonance imaging (MRI)-targeted transrectal ultrasound fusion (MRI-fusion) biopsy has shown increased detection of clinically significant PCa and reduced identification of clinically insignificant PCa (2), where the WPG segmentation is critical to enabling the MRI-fusion biopsy (3). Also, prostate volume measurement *via* WPG segmentation can be used to quantify the progression of benign prostatic hyperplasia (1) and to assist surgical planning (4).

Manual segmentation of WPG is time-consuming and laborious and commonly suffers from inter-rater variability (5), making it inadequate for large-scale applications (6). Deep learning (DL) (7–10) has increasingly been utilized for the automatic segmentation of WPG. Zhu et al. (11) proposed a deeply supervised convolutional neural network (CNN) using convolutional information to segment the prostate from MR images. Cheng et al. (8) developed a DL model with holistically nested networks for prostate segmentation on MRI. Jia et al. (12) proposed an atlas registration and ensemble deep CNN-based prostate segmentation. In addition, attentive DL (13) models were introduced to enhance DL by paying attention to the particular regions of interest in an adaptive way and thus have achieved better segmentation performance than other DL-based models. However, to the best of our knowledge, the evaluation of these methods was currently limited by a relatively small sample size, ranging from tens to hundreds of MRI scans. It is relatively expensive to create large, continuous samples with manual segmentation of WPG, which limits the ability to test the DL models in a clinical setting.

In this paper, we evaluated a previously developed DL-based automatic segmentation model, deep attentive neural network (DANN) (13), using a large, continuous cohort of prostate 3T MRI scans acquired between 2016 and 2020. The WPG segmentation by DANN was evaluated both quantitatively and qualitatively. The quantitative evaluation was performed by using an independent testing set with manual segmentation as a ground truth on a small dataset (n = 100). The dice similarity coefficient (DSC) (14) was used to measure the segmentation performance, compared with other baseline DL methods. For qualitative evaluation, the segmentation performance was evaluated by two abdominal radiologists independently *via* visual grading since the ground-truth manual segmentation was not available for the large cohort (n = 3,210). Inter-rater agreement between the two radiologists was evaluated to check the consistency of the visual grading. We further investigated the segmentation on different anatomical locations (i.e., apex, midgland, and base) as a secondary analysis. Finally, we conducted the volume measurement using DANN-based segmentation on a small cohort (n = 50) (DANN-enabled volume measurement) and compared it with the manual volume measurement.

## Materials and Methods

### MRI Datasets

With approval from the institutional review board (IRB), this retrospective study was carried out in compliance with the United States Health Insurance Portability and Accountability Act (HIPAA) of 1996. After excluding MRI scans with severe artifacts and patients with prior surgery history and Foley catheter, a total of 3,695 MRI scans, acquired on 3 T scanners (Skyra, Prisma, and Vida, Siemens Healthineers, Erlangen, Germany), from January of 2016 to August of 2020, were included in the study. Axial and coronal T2-weighted (T2W) Turbo spin-echo (TSE) images were used. **Table 1** shows the characteristics of the T2W MRI scan in the study.

**Table 1:** T2-weighted TSE MRI sequence parameters in the study.

View	Axial	Coronal
Matrix size	320 × 320	320 × 320
Flip angle	160°	147°
Resolution	0.625 × 0.625 × 3.6	0.625 × 0.625 × 3.6
Field of view (mm^2^)	200 × 200	200 ×200
Repetition time (ms)	3,000–7,480	2,880–7,200
Echo time (ms)	97–112	97–109
Number of slices	20	20
Scan time (s)	200	200

ms, millisecond; s, second; mm, millimeter.

Out of 3,695 3T MRI scans, 335 MRI scans (9%) were used as a training set, and the remaining 3,360 (91%) MRI scans were used as a testing set. Training and testing datasets were randomly chosen from the whole dataset. The testing set included a qualitative evaluation set (n = 3,210), a quantitative evaluation set (n = 100), and a volume measurement evaluation set (n = 50). **Table 2** shows the data characteristics for each dataset. Training, quantitative, and volume measurement evaluation sets required manual prostate contours as the segmentation reference standard. The manual annotation was prepared by an abdominal radiologist (MQ) with more than 5 years of experience in the interpretation of prostate MRI. In the training set, prostate contours were drawn on all axial T2W images from all MRI scans and on four mid-coronal T2W images (8th to 11th out of twenty slices) from a subset of 100 MRI scans. In the quantitative and volume measurement evaluation sets, prostate contours were drawn on all axial T2W images.

**Table 2:** Data characteristics in the training, qualitative, and quantitative evaluation.

		Training dataset	Qualitative evaluation dataset	Quantitative evaluation dataset	Volume evaluation dataset
Number of MRI scans		335	3,210	100	50
Number of patients with endorectal coil		3	84	0	0
MRI scans with different vendors	Skyra	295	2,806	93	45
	Prisma	10	145	4	3
	Vida	30	259	3	2

### DL-Based Whole Prostate Gland Segmentation Model

**Figure 1:**
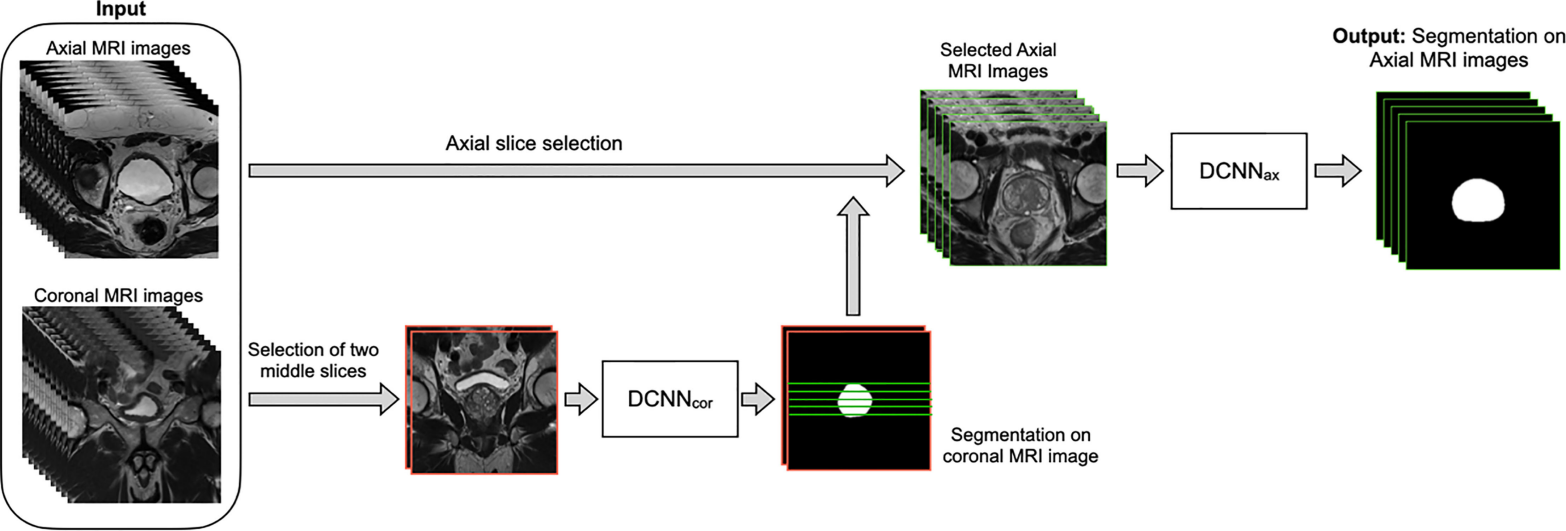
The overall workflow of the automatic WPG segmentation with DANN. Both axial and coronal T2W images were used as input, where the coronal images were used to assist the selection of certain axial images containing the prostate gland. DANN_cor_ was firstly performed on the two middle coronal images, indicated by images with the red border. Next, green lines selected by the prostate segmentation on the coronal images were used to determine the selection of axial slices (images with green borders). Once the axial images were selected, DANN_ax_ was performed on the axial MRI slices for the segmentation of WPG.

**Figure 1** shows the overall workflow of the automatic WPG segmentation with DANN (13). We added the segmentation on the coronal plane to assist the selection of axial slices, reducing the inference time of segmentation on the axial plane. During the testing, the workflow went through the following steps. First, DANN_cor_, responsible for segmenting coronal slices, was adopted to segment the prostate on the two-middle coronal images (9th and 10th slices out of twenty slices) for each MRI scan in the entire testing set. The segmented coronal images were used to automatically select the axial T2W images that contained the prostate gland. This would provide proper through-plane coverage of the prostate in the axial slices. Next, DANN_ax_ was used to perform the WPG segmentation on the selected axial T2W images for each MRI scan in all the testing sets.

Both DANN_ax_ and DANN_cor_ were trained independently using the training set (n = 335). First, a subset of the training data (n = 100) was used for training of DANN_cor_, and four-middle coronal slices (8th to 11th slices out of twenty slices) were used to make use of as many samples as possible. Once the initial training of DANN_cor_ was finished, two middle coronal slices were used as input to DANN_cor_ for the rest of the training data. The segmented coronal slices by DANN_cor_ were used to select certain axial slices, and DANN_ax_ was trained using all the selected axial slices in the entire training set. Training and inferencing were conducted on a desktop computer with a 64-Linux system with 4 Titan Xp GPU of 32 GB GDDR5 RAM. All the networks were trained with stochastic gradient descent as the optimizer, with binary cross-entropy as the loss function. PyTorch was used to implement all the DL networks. The models were initially trained using 80% of the training dataset, and the rest of the training dataset was used as the validation dataset. After the optimal hypermeters were found, we retrained the models using the whole training dataset. The learning rate was initially set to 2.5e-3. All the networks were trained for 100 epochs with batch size 12.

### Evaluation of Segmentation Performance

#### Qualitative Evaluation of Segmentation Performance

**Figure 2:**
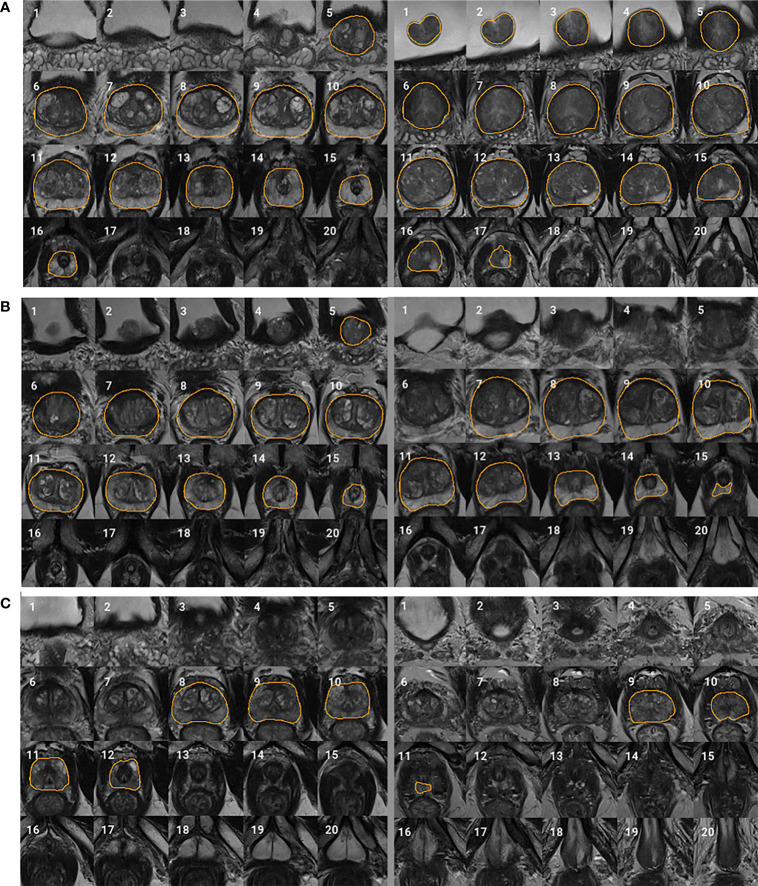
Typical examples for each visual grade. Rows **(A–C)** represent two segmentation examples with visual grades 3 (excellent), 2 (acceptable), and 1 (unacceptable), respectively. Slices 1–20 represent MRI slices from superior to inferior. Regions encircled by organ boundary are the prostate whole gland.

We adopted the visual grading, similar to (15), to qualitatively evaluate the WPG segmentation. Two abdominal radiologists (MQ and CS; each has over five years of experience in prostate MRI interpretation) assigned a visual grade, ranging from 1 to 3, to evaluate the segmentation performance, focusing on the whole prostate and sub-portions of the prostate (e.g., apex, midgland, and base). 1, 2, and 3 indicate unacceptable, acceptable, and excellent segmentation performance, respectively. **Table 3** shows the details when assigning the visual grade. Typical examples associated with each visual grade are shown in **Figure 2**. The readers independently ranked the segmentation quality. In addition, inter-rater reliability was assessed. To further investigate the segmentation at sub-portions of the prostate, we performed the sub-analysis for MRI scans without excellent segmentation performance agreed by both radiologists. Also, the segmentation performance for MRI scans with and without endorectal coil (ERC) was compared.

**Table 3:** Description of each visual grade for qualitative segmentation evaluation.

Score	Visual scoring description
3	The segmentation is excellent. The vast majority (>90%) of the prostate region has been correctly segmented, and the percentage of prostate slices with the failure segmentation is less than 10%.
2	The segmentation is acceptable. Most of the region (>70%) is correctly segmented, and the percentage of prostate slices that the method fails to segment is less than 30%.
1	The segmentation is unacceptable. More than 30% of the prostate region has been not correctly segmented or wrongly segmented, or the percentage of prostate slices that the method fails to segment is larger than 30%.

#### Quantitative Evaluation of Segmentation Performance

3D DSC (16) was used to quantitatively evaluate and compare the segmentation performance in the quantitative evaluation set (n = 100). The manual segmentations (M) were prepared by the radiologist on all axial slices as ground truths. DSC measures the overlapping between M and method-based (N) segmentation of the WPG volume and can be formulated as:


(1) $$DSC=\frac{2\left|M\cap M\right|}{\left|M\right|\cup \left|N\right|},$$

where ∩ and ∪ indicate the intersection and union, respectively.

#### Evaluation of Volume Measurement

We further evaluated the performance of DANN-enabled volume measurements. After the radiologist manually drew the WPG contour on all axial slices, Pyradiomics (17) was used to calculate the prostate volume in the volume measurement evaluation set (n = 50). The prostate volume from the DANN-based segmentation was compared with the manual volume measurement. The Bland–Altman plot (18) was used to analyze the agreement between manual and DANN-enabled WPG volume measurements.

### Statistical Analysis

Mean and standard deviation were used to describe the distribution of DSC. The quantitative segmentation performance difference between the DANN and the baselines was compared using a paired sample t-test (19). p values < 0.05 were considered statistically significant. Inter-rater reliability between two radiologists was measured by using the κ statistic (20). The relationship between the value of κ and inter-rater reliability is listed as below, κ < 0: pool agreement; 0 < κ < 0.2: slight agreement; 0.21 < κ < 0.4: fair agreement; 0.41 < κ < 0.6: moderate agreement; 0.61 < κ < 0.8: substantial agreement; 0.81 < κ < 1.0: almost perfect agreement.

## Results

### Qualitative Evaluation of WPG Segmentation

**Figure 3:**
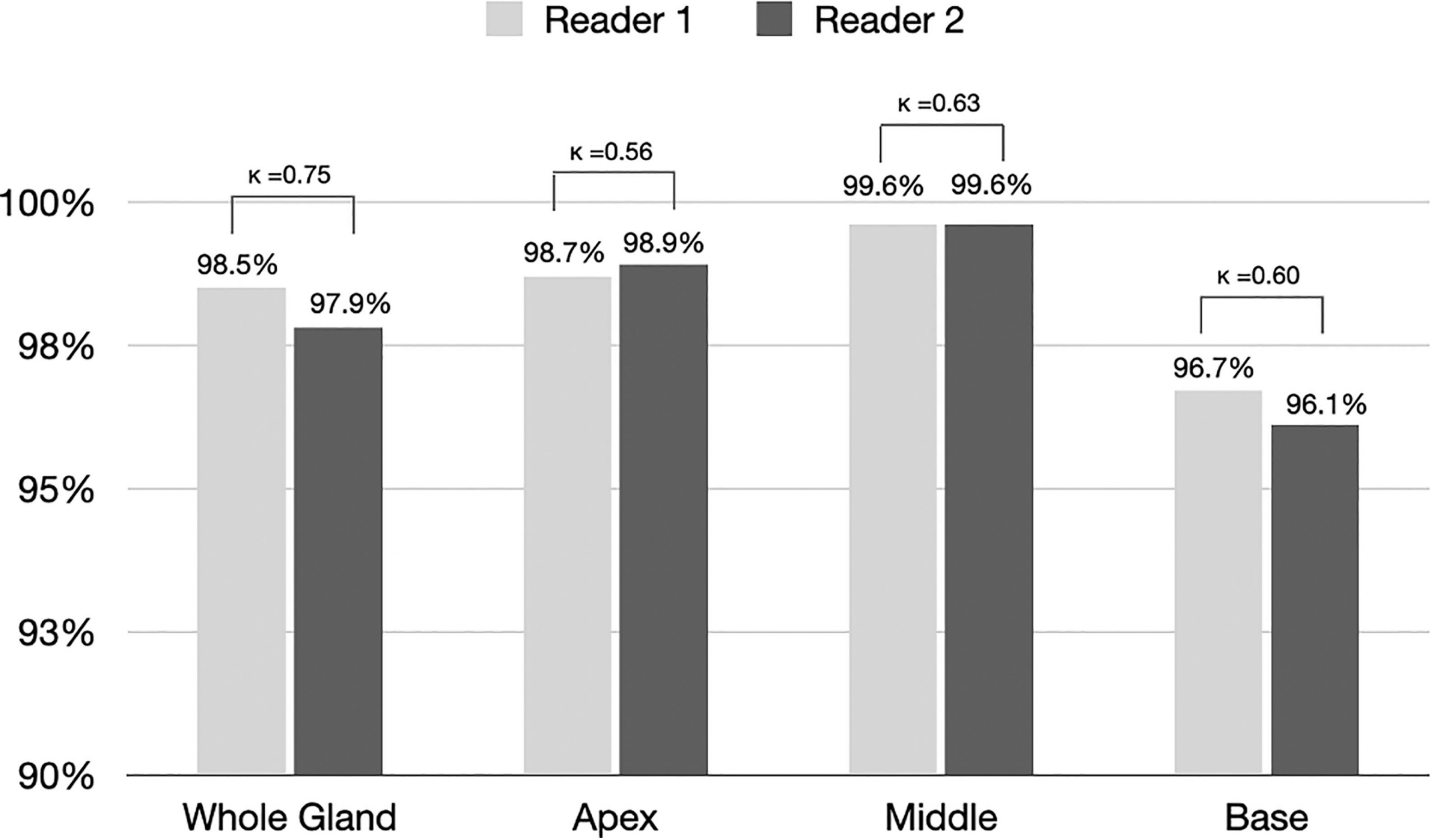
The proportion of segmentation with acceptable or excellent performance evaluated by radiologists 1 and 2 among all MRI scans (n = 3210). Kappa statistics between the two readers were also provided in the figure.

**Figure 3** shows the proportion of acceptable or excellent segmentation quality in all MRI scans on the qualitative evaluation set at the whole prostate, or each sub-portion (apex, midgland, or base) of the prostate. The DANN method exhibited an acceptable or excellent segmentation performance in more than 96% of the MRI scans on the whole prostate or each sub-portion of the prostate. The segmentation at the midgland portion had achieved the best segmentation performance, while it performed the worst at the base portion.

#### Qualitative Evaluation and Inter-Rater Variability for WPG Segmentation

For WPG segmentation, 97.9% (n = 3,141) and 93.2% (n = 2,992) of the MRI scans were graded as having acceptable or excellent segmentation performance. **Table 4** includes the confusion matrix to show the inter-rater variability of the visual grading. Overall, two readers reached a substantial consensus on the visual grading in 95.8% of the patients (κ = 0.74). When readers differed on the grading, the discrepancy in grading was less than one. A percentage of 94.6% of segmentation results were unanimously considered as acceptable or excellent. Moreover, 91.5% of the MRI scans (n = 2,861) were graded as having excellent segmentation performance according to the two radiologists. Unacceptable segmentation performance occurred only in 1.2% of the MRI scans (n = 39), agreed by the two radiologists.

**Table 4:** Confusion matrices between the visual grades assigned by two readers.

All	Reader 2				Kappa (κ)
Reader 1	Visual grade	1	2	3	Substantial agreement (κ =0.75)
	1	47 (1.5)	1 (0.0)	0 (0.0)	
	2	22 (0.7)	99 (3.1)	49 (1.5)	
	3	0 (0.0)	63 (2.0)	2,929 (91.3)	

Kappa coefficient (κ) is used to measure the inter-rater variability between the two readers.

#### Sub-Analysis of MRI Scans Without Excellent WPG Segmentation

**Figure 4:**
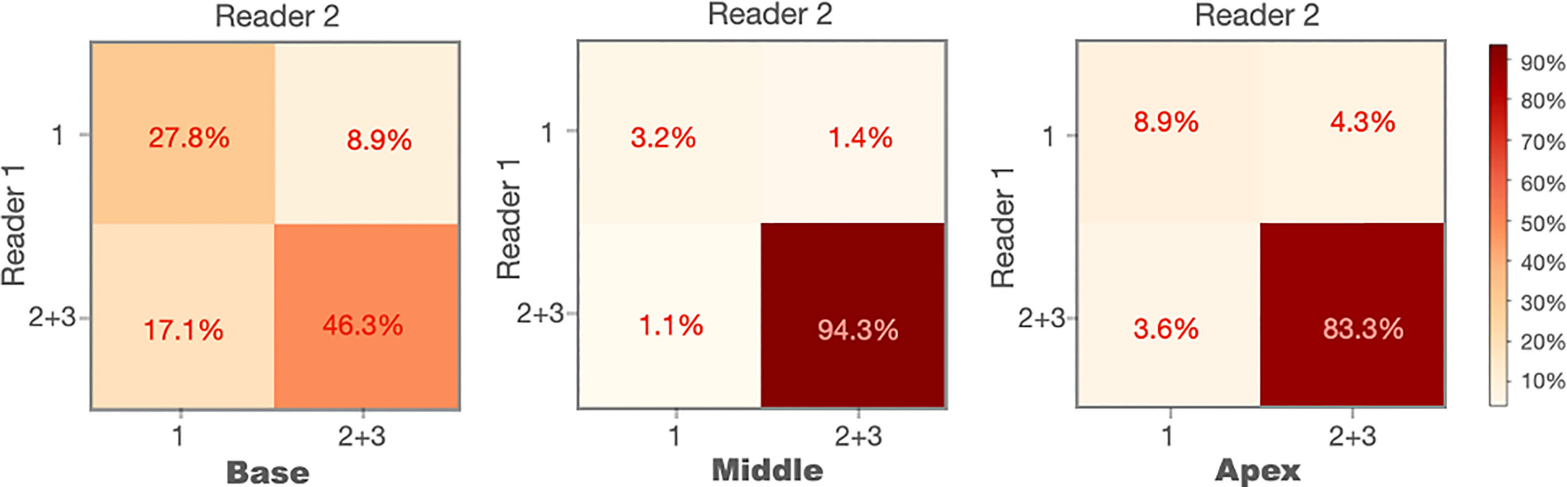
Confusion matrices of the prostate base, midgland, and apex for the cases without excellent segmentation (n = 281).

We conducted the sub-analysis related to each sub-portion of the prostate (apex, midgland, or base) when the WPG segmentation was not excellent. The MRI scans with excellent segmentation agreed by two readers were excluded (n = 2,929), and the rest of the MRI scans were used for the analysis (n = 281). **Figure 4** shows the confusion matrices of each sub-portion of the prostate on the rest of the MRI scans. A percentage of 46.3% of the MRI scans achieved the acceptable (or better) segmentation quality at the base slices, while 94.3% and 83.3% of the MRI scans achieved the acceptable (or better) segmentation quality at the midgland and apex slices.

#### Comparison Between MRI Scans With and Without ERC

**Figure 5:**
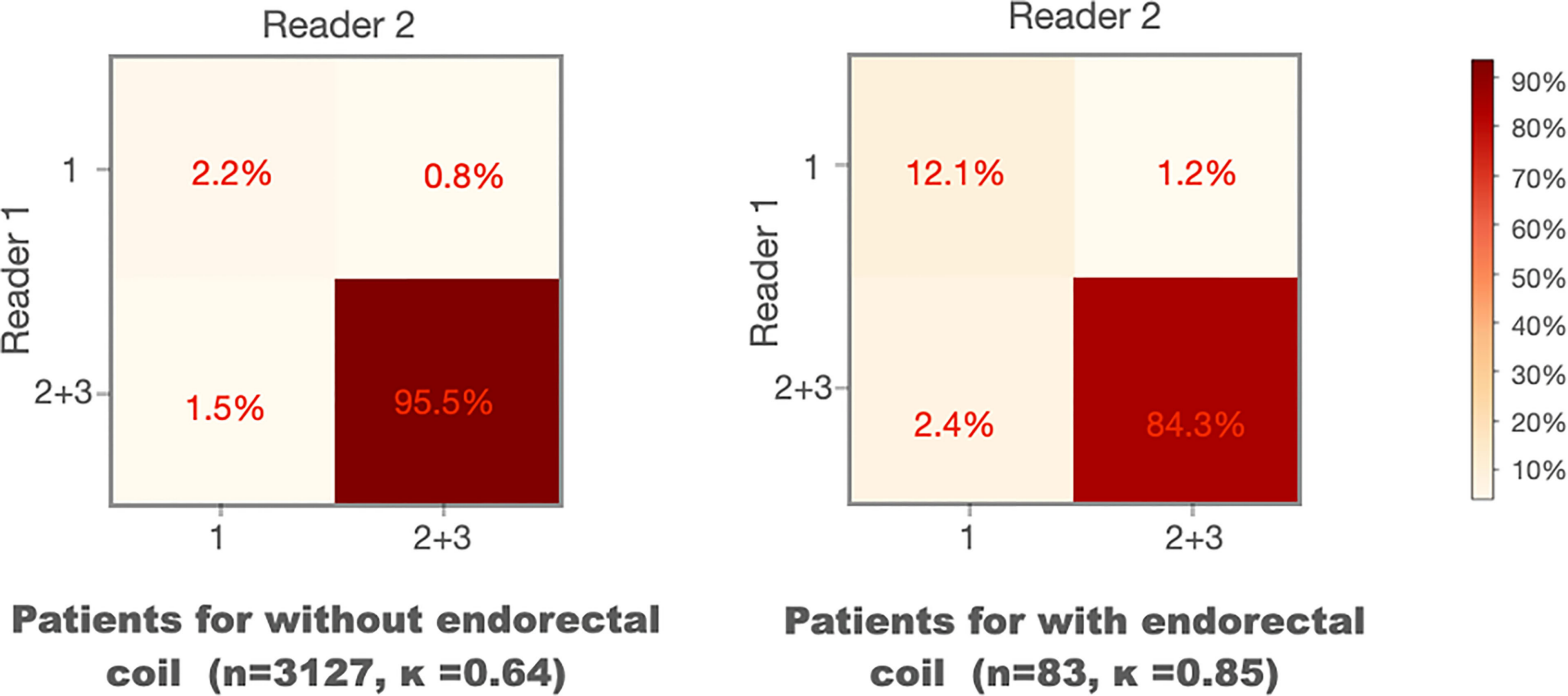
Confusion matrices of the visual grades of segmentation on MRI scans with and without endorectal coils. Kappa coefficient (κ) is used to measure the inter-rater variability between the two readers.

We compared the WPG segmentation quality for the MRI scans with and without ERC (21). **Figure 5** shows the confusion matrices of the visual grades of segmentation on MRI scans with and without ERC. There were substantial agreements (κ = 0.64 and 0.85) between the two radiologists on WPG segmentation of MRI scans with and without ERC. When considering the inter-rater agreement of WPG segmentation, DANN demonstrated acceptable WPG performance in more than 95.5% of MRI scans with ERC compared to 84.3% of those without ERC. MRI scans with ERC had a larger proportion of unacceptable WPG segmentation compared to those without ERC (12.1% vs. 2.2%).

### Quantitative Evaluation of WPG Segmentation

The quantitative performance of the DANN was compared to the other two baseline methods, including DeepLab v3+ (22) and UNet (23). **Table 5** shows the comparisons of DSCs between DANN and the baseline methods. The DANN achieved a DSC of 0.93, which was higher than those of DeepLab v3+ and UNet with significant differences (both p values < 0.05).

**Table 5:** Quantitative DSC comparisons with baseline methods.

Methods	DSC
Proposed method	0.93 ± 0.02
DeepLab v3+	0.92 ± 0.02 p < 0.05
UNet	0.91 ± 0.03 p < 0.05

### Evaluation of Volume Measurement

**Figure 6:**
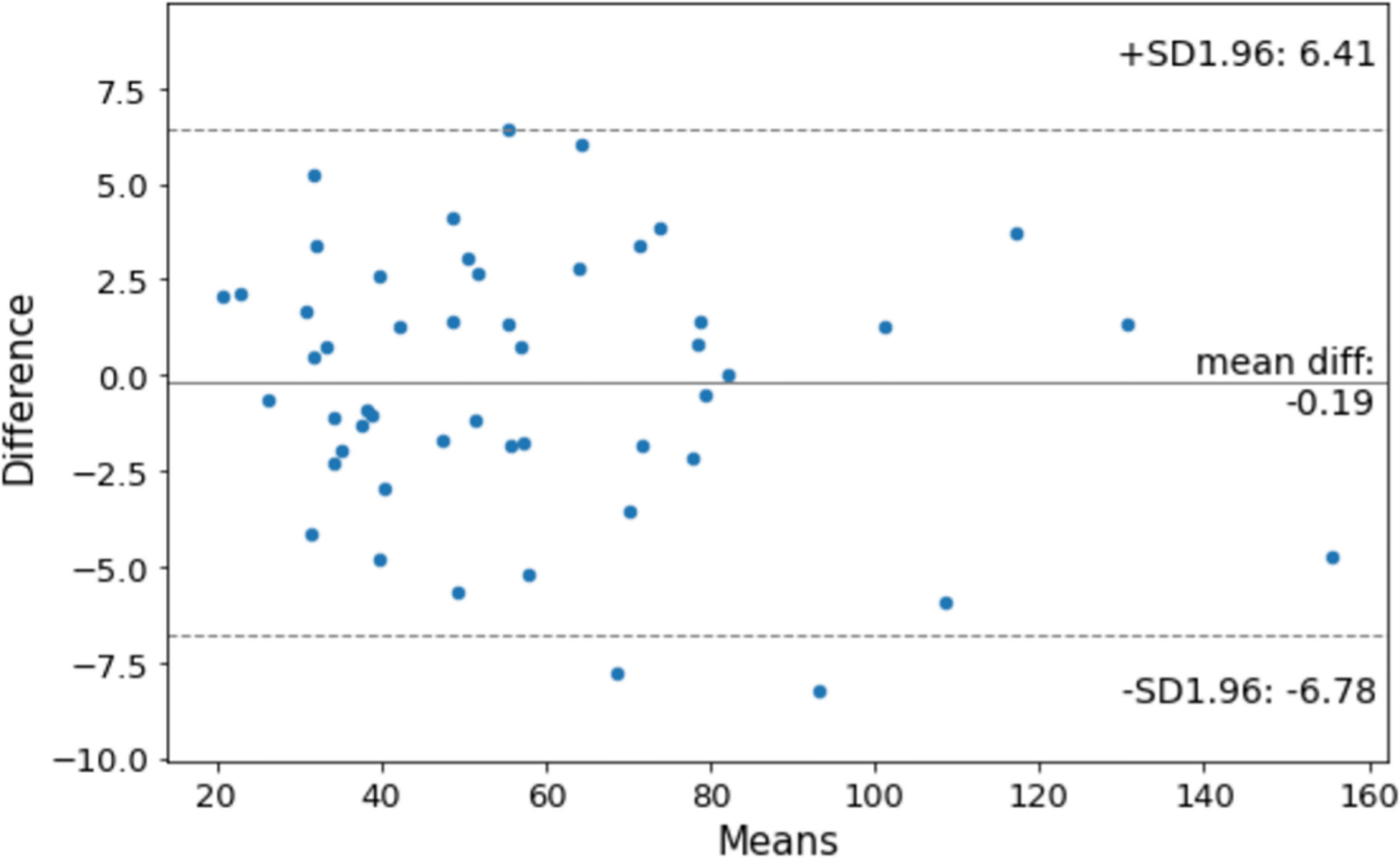
Bland–Altman plot to show the agreement between manual and DANN-enabled WPG volume measurements.

**Figure 6** shows the agreement between manual and DANN-enabled volume measurements in the Bland–Altman plot. The mean difference between the two-volume measurements was calculated as an estimated bias. Standard deviation (SD) of the differences, and 95% limits of agreement (average difference ± 1.96 SD) were calculated to assess the random fluctuations around this mean. A total of 48 out of 50 cases (96%) had the volume measurement differences within 95% limits of agreement, indicating that the manual and DANN-enabled volume measurements can be potentially used interchangeably.

## Discussion

A deep attentive neural network (13), DANN, for the automatic WPG segmentation was evaluated on a large, continuous patient cohort. In the qualitative evaluation, DANN demonstrated that the segmentation quality is either acceptable or excellent in most cases. Two radiologists exhibited a substantial agreement for the qualitative evaluation. In the quantitative evaluation, DANN exhibited a significantly higher DSC than the baseline methods, such as UNet and DeepLab v3+. Also, 96% of the testing cohort had volume measurement differences within 95% limits of agreement.

We found that DANN demonstrated worse segmentation performance at the prostate base than at the apex and midgland slices. This may be due to the fact that the anatomical structure of the prostate base is relatively more complex than other prostate portions. The prostate base is in continuity with the bladder and seminal vesicles, and thus the boundary may contain partial volume effects and mild movement artifacts.

We observed that the segmentation performance was somewhat limited when MRI scans were acquired with an ERC. We believe that this may be because there were only three MRI scans with ERC in the training dataset. A large training data with ERC may allow the model to learn representative features related to the prostate MRI with ERC. In addition, images often exhibit large intensity variation compared to the MRI scans without ERC as ERC is close to the prostate. This may require including an even larger training dataset to account for these intensity variations than those without ERC.

We refined DANN by adding the coronal segmentation to assist the selection of axial slices for WPG segmentation. With assistance from the coronal segmentation, the axial model conducted the segmentation only on the selected axial slices instead of applying it to all axial slices, which reduces the inference time. **Table 6** contains the inference time between the segmentation with and without coronal segmentation. The total inference time in a combination of coronal and axial slices was 25% less than the inference time without assisting the selection of axial slices (12.6 vs. 16.4 min). In addition, we observed that DSC was not different when adding the coronal segmentation in the quantitative evaluation.

**Table 6:** Inference time estimation and DSCs obtained with and without coronal segmentation assistance.

	Without coronal segmentation assistance	With coronal segmentation assistance
Overall inference time estimation in the qualitative evaluation	16.4 min (67,775)	12.6 min (45,713)
DSCs obtained in the quantitative evaluation	0.93	0.93

() indicates the total amount of MRI slices the method needed to segment.

Compared with quantitative evaluation, qualitative evaluation includes unique characteristics and benefits. The DSC-based evaluation often overlooks the segmentation performance on small regions when they were combined with larger regions. Prostate at apex or base slices is relatively smaller than the one in the middle, and therefore, the quantitative evaluation may not be sensitive enough to illustrate limitations at these locations when 3D DSC is used for the evaluation. Also, the DSC-based evaluation is not directly associated with clinical implications, while qualitative evaluation categorized the segmentation results based on the quality to which segmentation can be acceptable clinically.

Our study still has a few limitations: 1) the MRI scans in this study were acquired from three 3T MRI scanners at a single medical center. Prostate MRI sequence parameters are generally well-standardized by the Prostate Imaging–Reporting and Data System (PI-RADS) guidelines (24), but future studies would include testing DANN at multiple institutions. 2) The inter-rater variability was tested between two radiologists. We will include more radiologists to evaluate comprehensive inter-rater variability. 3) Large GPU memory was required during the training and testing since DANN included the spatial attention mechanism that caused considerable computational complexity. In the future, we will explore the criss-cross attention module (25) that uses the contextual information of all the pixels on the criss-cross path for each pixel, which has shown the potential to reduce the GPU memory.

## Conclusion

Our study showed that the proposed deep learning-based prostate segmentation (DANN) could generate segmentation of the prostate with sufficient quality in a consistent manner when a large, continuous cohort of prostate MRI scans was used for evaluation. The qualitative evaluation conducted by two abdominal radiologists showed that 95% of the segmentation results were either acceptable or excellent with a great inter-rater agreement. In the quantitative evaluation, DANN was superior to the state-of-art deep learning methods, and the difference between manual and DANN-enabled volume measurements was subtle in most cases. The method has a great potential to serve as a tool to assist prostate volume measurements, and biopsy and surgical planning in a clinically relevant setting.

## Data Availability Statement

The raw data supporting the conclusions of this study will be made available by the authors in accordance with institutional management and sharing policy. 

## Ethics Statement

The studies involving human participants were reviewed and approved by the Institutional Review Board of UCLA. The ethics committee waived the requirement of written informed consent for participation.

## Author Contributions

Conceptualization, YL and KS. Methodology, YL. Software, YL. Validation, QM and CS. Formal analysis, YL and KS. Investigation, YL and KS. Resources, KS and SR. Data curation, YL. Writing—original draft preparation, YL and KS. Writing—review and editing, YL, KS, SR, GY, QM, CS, and DN. Visualization, YL. Supervision, KS and SR. Project administration, KS. Funding acquisition, KS and GY. All authors contributed to the article and approved the submitted version.

## Funding

This work was supported in part by the National Institutes of Health R01-CA248506 and funds from the Integrated Diagnostics Program, Departments of Radiological Sciences and Pathology, David Geffen School of Medicine, UCLA. This study was also supported in part by the British Heart Foundation (Project Number: TG/18/5/34111, PG/16/78/32402), the European Research Council Innovative Medicines Initiative (DRAGON, H2020-JTI-IMI2 101005122), the AI for Health Imaging Award (CHAIMELEON, H2020-SC1-FA-DTS-2019-1 952172), and the UK Research and Innovation Future Leaders Fellowship (MR/V023799/1).

## Conflict of Interest

The authors declare that the research was conducted in the absence of any commercial or financial relationships that could be construed as a potential conflict of interest.

## Publisher’s Note

All claims expressed in this article are solely those of the authors and do not necessarily represent those of their affiliated organizations, or those of the publisher, the editors and the reviewers. Any product that may be evaluated in this article, or claim that may be made by its manufacturer, is not guaranteed or endorsed by the publisher.
